# Microsporidial keratoconjunctivitis – first outbreak in Japan

**DOI:** 10.1186/s12879-023-08767-y

**Published:** 2023-11-01

**Authors:** Masafumi Uematsu, Yasser Helmy Mohamed, Mao Kusano, Daisuke Inoue, Kohei Harada, Diya Tang, Takashi Kitaoka, Kenji Yagita

**Affiliations:** 1 Department of Ophthalmology and Visual Sciences, Graduate School of Biomedical Sciences, Nagasaki University, 1-7-1 Sakamoto, Nagasaki, 852-8501 Japan; 2 Department of Parasitology, The National Institute of Infectious Diseases, Tokyo, Japan

**Keywords:** Microsporidial keratoconjunctivitis, Anterior segment optical coherence tomography (AS-OCT), In vivo confocal biomicroscopy (IVCM), Polymerase chain reaction (PCR), *Vittaforma corneae*

## Abstract

**Background:**

Most cases of microsporidial keratoconjunctivitis are found in the Southern hemisphere. Our purpose was to investigate the first outbreak of microsporidial keratoconjunctivitis in Japan among healthy, immunocompetent soccer players from the same team during a 1-month period.

**Case presentation:**

This study is an observational case series. The medical records were analyzed for five cases with microsporidial keratoconjunctivitis who presented within September 2022. All five cases were males between 28 and 36 years old. These previously healthy individuals belonged to the same football team. Their eyes were considered susceptible to contaminated water or dirt from the turf at game and practice sites. All cases involved unilateral conjunctivitis, with scattered round white lesions that showed positive fluorescein staining in the corneal epithelium. All cases experienced diminution of vision in the affected eye. In three cases, direct smears showed spores of approximately 2–3 μm in diameter. Polymerase chain reaction (PCR) analysis of corneal scrapes revealed partial amplification of microsporidial 18 S ribosomal RNA gene in four cases. Sequences of PCR products from all four cases showed 100% identity with strains of *Vittaforma corneae* previously reported from an outbreak in Singapore. All cases were treated with topical therapy, including voriconazole, fluorometholone, and levofloxacin. Four eyes underwent corneal scraping. After treatment, all eyes healed without residual opacities.

**Conclusions:**

Only a few sporadic case reports of this disease have previously been reported in Japan. We detected *V. corneae* in our case series, representing what appears to be the first outbreak of microsporidial keratoconjunctivitis in Japan. Exposure to contaminated water or soil, in addition to inadequate sanitary facilities, represents a potential source of infection. Further investigations to clarify the characteristics of microsporidia seem warranted.

**Supplementary Information:**

The online version contains supplementary material available at 10.1186/s12879-023-08767-y.

## Background

Microsporidia are unicellular, obligate intracellular, eukaryotic parasites belonging to the phylum *Microspora* in the subkingdom Protista. These organisms can infect both vertebrates and invertebrates [1]. Microsporidiosis occurs worldwide, with the reported prevalence varying based on geographical and demographic characteristics, along with the diagnostic methods used for diagnosis. In humans, microsporidia are primarily opportunistic pathogens seen most frequently among individuals with acquired immunodeficiency syndrome, organ transplant recipients, and patients receiving immunosuppressants, but can also occur in immunocompetent patients [2]. Microsporidia have been implicated in infections of the intestines, eyes, sinuses, lungs, muscles, kidneys, and central nervous system [3]. Besides infections of the digestive tract, ocular disease is the second most common clinical manifestation of microsporidiosis. In the eye, the most frequent presentations are superficial epithelial keratoconjunctivitis and deep stromal keratitis [4].

In the early 2000s, case series of microsporidia keratoconjunctivitis (MKC) were reported among immunocompetent individuals from Singapore and India. Increasing reports from Singapore, Hong Kong, Thailand, and India have indicated the endemic nature of this pathology [5]. However, only a few sporadic cases have been reported in Japan [6, 7]. In 2022, we encountered a series of cases involving unilateral microsporidial epithelial keratitis in healthy, soccer players from the same team with a one-month period. To the best of our knowledge, this represents the first case series of MKC to be reported from Japan. We describe this case series herein.

## Case presentation

This study was approved by the institutional review board of Nagasaki University Hospital and adhered to the tenets of the Declaration of Helsinki. Informed consent to publish this case report was obtained from all individual participants included in the study.

Cases with unilateral keratoconjunctivitis initially treated by general ophthalmologists were referred to the cornea clinic in the Department of Ophthalmology, Nagasaki University Hospital in September 2022. All cases were evaluated by cornea specialists using comprehensive ophthalmic examinations, including history taking, symptom analysis, best-corrected visual acuity (BCVA) measurements, slit lamp biomicroscopy, anterior segment optic coherence tomography (AS-OCT) and in vivo confocal biomicroscopy (IVCM). Corneal epithelial scraping was also performed where possible and specimens were examined by staining and polymerase chain reaction (PCR) analysis. After epithelial scraping, soft contact lenses (SCL) were applied to the affected corneas. Treatment included voriconazole eye drops 6 times/day, fluorometholone 0.1% and levofloxacin eye drops 4 times/day. All cases were followed-up until full recovery, with complete ophthalmic evaluation. The duration of the disease onset ranged between 1.5: 3 weeks.

### Laboratory diagnosis

Corneal scrapings were obtained from consenting cases for the detection of microsporidia. After instillation of a topical anesthetic agent (0.5% proparacaine hydrochloride), superficial corneal scraping was performed using a sterile spatula under microscopy. Smearing and inoculation of various media was performed immediately at the eye clinic. Slides and media were then sent to the laboratory. The scraping was smeared on two glass slides and subjected to direct microscopic examination after staining with 10% potassium hydroxide with Calcofluor White (fluorescent brightener; Sigma, USA) and Gram stain.

### PCR and DNA sequencing

Total DNA was extracted from the corneal scraping using a Gene clean® II kit (MP Biomedicals, USA). Briefly, a small piece of the corneal scraping was lysed in 200 μl of 10% SDS containing protease K (Qiagen, USA) at 70°C for 2 h. The lysate was pretreated with an equal volume of phenol/chloroform solution. After centrifugation at 10,000 rpm for 3 min, 100 μl of the lysate was applied to the DNA purification procedure using the Gene clean® II kit. Finally, DNA was eluted in 20 μl of TE buffer. The primer set comprising PMP1: 5’-CACCAGGTTGA TTCTGCCTGAC-3’ and msprv1: 5’-GTTGAGTCAAATTAAGCCGCA(C/T) A-3’ (newly designed in the present study) was used to amplify a partial region of approximately 750 bp of the small subunit ribosomal RNA gene of microsporidia. The program of PCR was as follows: 95 °C for 3 min in pre-heating, then a cycle of 94 °C for 30 s, 65 °C for 1 min, and 72 °C for 1 min, repeated 40 times, with final extension at 72 °C for 10 min. PCR products were directly sequenced. The resulting sequences were submitted to the basic local alignment search tool (BLAST) in NCBI for comparison of sequences with the NCBI database.

The demographic characteristics and clinical symptoms of all cases are summarized in Table 1. All cases were males between 28 and 36 years old (median age, 33 years). All cases initially reported a unilateral foreign body sensation and redness, which were treated by general ophthalmologists as viral conjunctivitis. This treatment proved ineffective, and symptoms persisted or exacerbated. The cases were therefore referred to the Cornea section in our department.

All cases were soccer players from the same team. None had any history of systemic disease that could affect the general health condition. One case (Case 1) had a history of mild ocular trauma, three cases were daily users of contact lenses (CLs), and one case (Case 5) had no history of trauma or CL use.

**Table 1:** The demography and clinical symptoms of all patients

Case	Age	Sex	Eye	Trauma/ CL	Affected Eye BCVA	Symptoms
1	36	M	Lt	Lt trauma	0.3	Redness, FB sensation, diminution of vision
2	31	M	Lt	CL daily use	0.1	Redness, pain, diminution of vision
3	30	M	Rt	CL daily use	0.3	Pain, lid swelling, diminution of vision
4	33	M	Rt	CL daily use	0.7	Redness, FB sensation, discharge, diminution of vision
5	28	M	Lt		1.2	FB sensation, discharge

M = male; Lt = left; Rt = right; CL = contact lens; BCVA = best corrected visual acuity; FB = foreign body

**Fig. 1:**
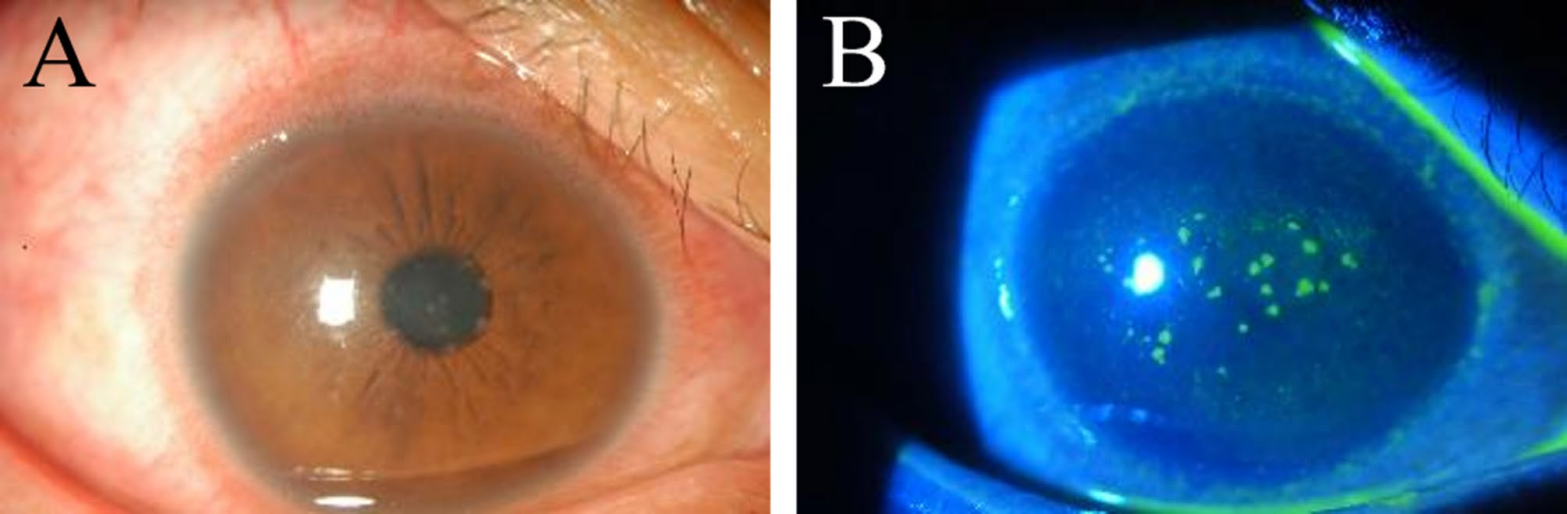
The cornea shows diffuse multifocal, coarse epithelial punctate lesions (**A**) that stain positively with fluorescein (**B**)

Clinical signs, laboratory investigations, treatment and outcomes for all cases are summarized in Table 2. Slit lamp biomicroscopy revealed that all cases had unilateral diffuse or paracentral (Case 5) multifocal, coarse epithelial punctate lesions that showed positive results for fluorescein staining (Fig. 1). Cases 1 and 2 exhibited mild anterior chamber inflammation in the form of mild flare and few cells with endothelial keratic precipitants. All cases had diffuse conjunctival inflammation and redness, and three cases (Cases 2–4) showed papillae. Four cases experienced diminution of vision in the affected eye.

**Table 2:** Clinical signs, laboratory investigations, treatment and outcome of all cases

	*Signs*	*AS-OCT*	*IVCM*	*Staining*	*PCR*	*Treatment*	*Outcome*
1	Diffuse multifocal, coarse epithelial lesions with AC inflammation, diffuse conjunctivitis	Epithelial hyperreflective spots with diffuse stromal infiltration	NA	NA	+ve	Epithelial scrapping and SCL + Voriconazole drops 6 times/day Fluorometholone 0.1% and Levofloxacin drops 4 times/day	Complete corneal epithelial healing with no residual opacity, BCVA = 1.0
2	Diffuse multifocal, coarse epithelial lesions with AC inflammation, papillary conjunctivitis	Epithelial and anterior stromal infiltration	Spores in epithelium and anterior stroma	Gram +ve CFW + ve	+ve	Epithelial scrapping and SCL + Voriconazole drops 6 times/day Fluorometholone 0.1% and Levofloxacin drops 4 times/day	Complete corneal epithelial healing with no residual opacity, BCVA = 1.2
3	Central multifocal, coarse epithelial lesions with no AC inflammation, papillary conjunctivitis	Epithelial and anterior stromal infiltration	Spores in epithelium and anterior stroma	Gram +ve CFW + ve	+ve	Epithelial scrapping and SCL + Voriconazole drops 6 times/day Fluorometholone 0.1% and Levofloxacin drops 4 times/day	Complete corneal epithelial healing with no residual opacity, BCVA = 1.2
4	Central multifocal, coarse epithelial lesions with no AC inflammation, papillary conjunctivitis	Epithelial and anterior stromal infiltration	Spores in epithelium and anterior stroma	Gram + ve CFW + ve	+ve	Epithelial scrapping and SCL + Voriconazole drops 6 times/day Fluorometholone 0.1% and Levofloxacin drops 4 times/day	Complete corneal epithelial healing with no residual opacity, BCVA = 1.2
5	Diffuse multifocal, coarse epithelial lesions with no AC inflammation, diffuse conjunctivitis	Epithelial and anterior stromal infiltration	NA	NA	NA	Voriconazole drops 6 times/day Fluorometholone 0.1% and Levofloxacin drops 4 times/day	Complete corneal epithelial healing with no residual opacity, BCVA = 1.2

AC = anterior chamber; AS-OCT = anterior segment optical coherent tomography; IVCM = in vivo confocal biomicroscopy; PCR = polymerase chain reaction; NA = not applicable; CFW = Calcofluor White stain; SCL = soft contact lens; BCVA = best corrected visual acuity

### Imaging and laboratory findings

**Fig. 2:**
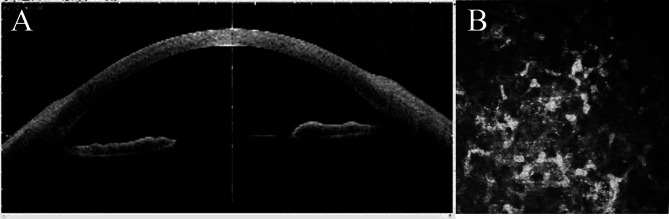
Anterior segment optical coherence tomography shows diffuse infiltration of the corneal stroma (**A**). In vivo confocal biomicroscopy shows hyper-reflective, pinpoint ovoid spores in and between epithelial cells (**B**)

On AS-OCT, the lesions appeared as hyperreflective spots mainly related to the epithelial layers, some of which were slightly raised above the surrounding surface. In addition, anterior stromal infiltrations were seen in the corneas of all affected eyes, with diffuse stromal edema (Fig. 2A). In IVCM, hyper-reflective, pinpoint ovoid spores were detected in and between epithelial cells and in the anterior stromal layers in three cases (Fig. 2B).

**Fig. 3:**
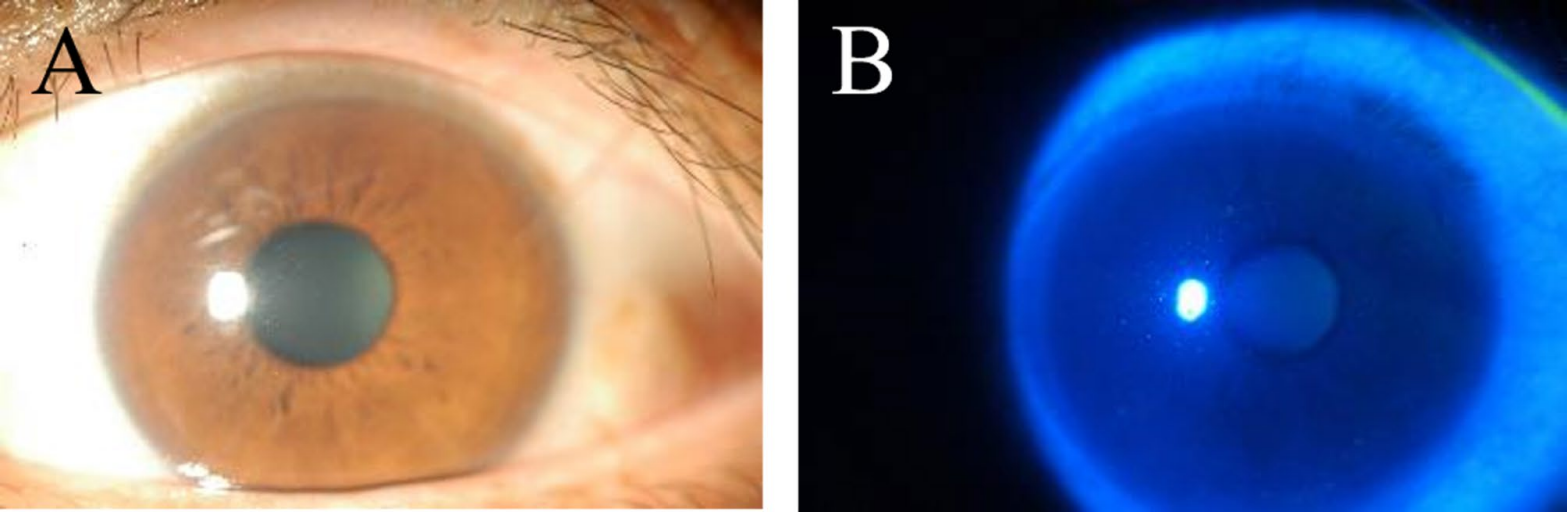
Complete corneal epithelial healing with no residual opacity within a few weeks after initiation of treatment (**A**) with negative fluorescein staining (**B**)

**Fig. 4:**
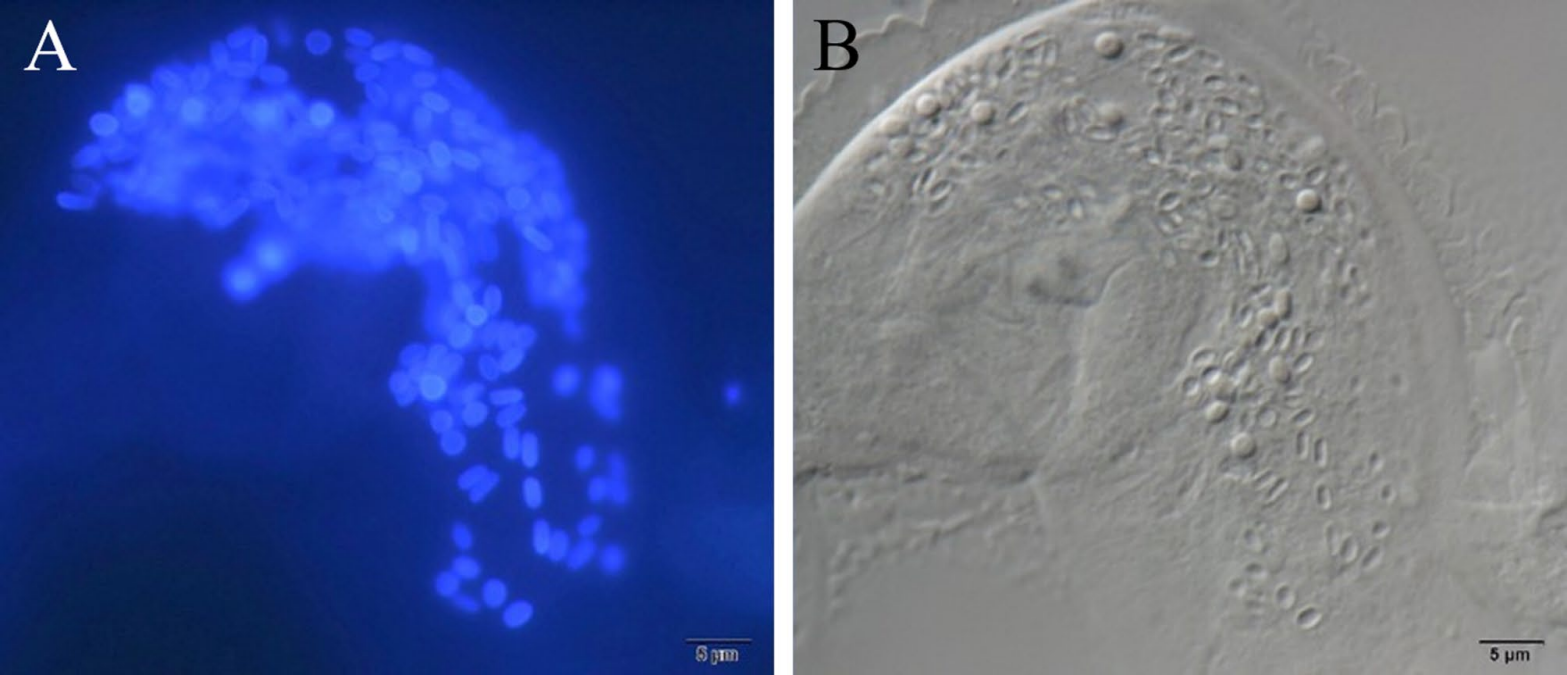
Microsporidial spores in the corneal scraping. (**A**) Calcofluor White staining shows numerous spores in the tissue. (**B**) A differential interference contrast image of the same specimen

Complete corneal epithelial healing with no residual opacity was achieved in all cases within a few weeks after initiating treatment, as confirmed by negative fluorescein staining (Fig. 3). In microscopy with differential interference contrast imaging, Gram staining, and Calcofluor White staining, round-or-oval, microsporidial spore-like structures were detected in three of the four smear samples. Figure 4 shows multiple spores in the corneal tissue of Case 2. Mean spore size was 2.9 × 1.6 μm (n = 30).

**Fig. 5:**
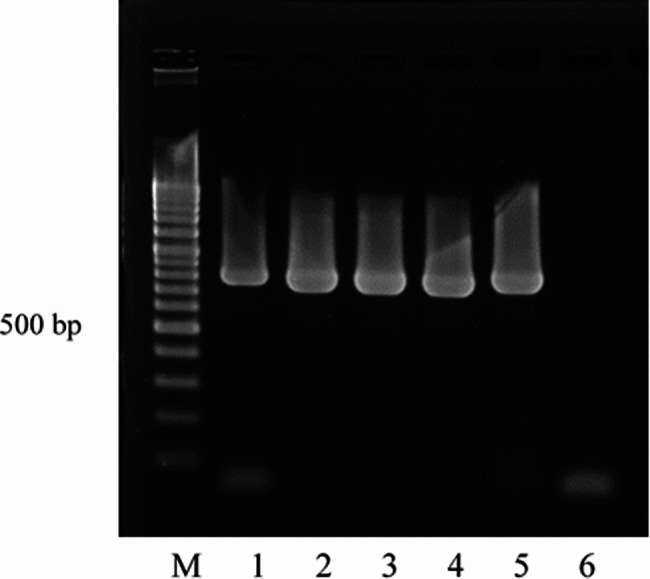
Agarose gel electrophoresis of PCR products from DNA extracts of corneal scrapings. Lanes 1–4: case 2, case 3, case 4 and case 5, respectively; Lane 5: *Encephalitozoon cuniculi* DNA as a positive control; Lane 6: negative control. All four cases show PCR products of the same size, approximat ely 750 bp

PCR of the DNA extracted from corneal scrapings successfully amplified approximately 750 bp of DNA, slightly smaller than that from *Encephalitozoon cuniculi* (Fig. 5). The sequences obtained from PCR products from the four cases were all identical. Full-length gels and blots are included in a Supplementary Information file. The results of BLAST analysis showed that the present sequences exhibited 100% homology with a partial sequence of the small subunit ribosomal RNA gene of the *Vittaforma corneae* HK1 strain (accession no. JX123131), which was identified from a previous microsporidial outbreak [8].

## Discussion and conclusions

Microsporidia are eukaryotic, obligate intracellular, spore–forming unicellular parasites that are known to infect vertebrates and invertebrates and have increasingly gained prominence in affecting humans over the past few decades [9]. Based on morphological and molecular studies, it has been reclassified recently as fungi [4]. The ocular manifestations of microsporidial infection include keratoconjunctivitis or stromal keratitis [10]. Only a small number of sporadic cases have been reported in Japan [6, 7, 11]. One case was treated with long-term steroid therapy for peripheral corneal ulcer complicated by rheumatoid arthritis. Opportunistic infection was suspected in a case of fungal keratitis combined with keratitis caused by microsporidia [6]. In a case of corneal opacity after corneal endothelial transplantation, keratitis due to microsporidia was diagnosed by transmission electron microscopy [11]. Finally, a Japanese patient developed MKC during a temporary return trip to Japan from Singapore [7]. To the best of our knowledge, this report offers the first description of an outbreak of MKC diagnosed in Japan.

Ocular microsporidiosis is not a new entity, but is being reported in increasing frequency, possibly due to increased environmental alterations and awareness and improved diagnostic ability. This infection can affect both immunocompromised and immunocompetent individuals. Most of the large series published have been from Asian tropical and semitropical regions [7, 12]. Nagasaki prefecture is situated in the southern part of Japan, with a humid extratropical climate. In August 2022, just prior to the MKC outbreak we described, mean temperature and total rainfall were 28.6 °C and 371.5 mm, respectively, in Nagasaki prefecture (Japan Meteorological Agency, https://www.jma.go.jp). These values were comparable to the average temperature of 27.6 °C and total rainfall of 339.4 mm in Singapore in April 2012, when an MKC outbreak occurred (Singapore National Environment Agency, https://www.nea.gov.sg) [9]. One possibility is that soil and/or water may have harbored pathogenic microsporidia in the humid subtropical climate of this area, especially in the summer season.

The exact mode of transmission for ocular microsporidiosis is not known. Contaminated water, in addition to inadequate sanitary facilities, is a potential source of infection. In Singapore, Loh et al. observed such correlations in 50% (62 of 124) of their patients engaged in outdoor activities, including soccer, golf, and trail biking, particularly after rainfall [13]. A few outbreaks of ocular microsporidiosis in rugby players have been reported, likely secondary to exposure to contaminated soil on the playing field [8, 14]. Minor trauma can cause direct injection of the organism onto the corneal epithelium following exposure to soil/muddy water harboring the spores [15]. Contact lenses were first reported in 2001 as a carrier of spores for one healthy individual in Singapore [16], followed by a larger series of 25 cases (21.1%) also from Singapore [13].

All of our cases were soccer players on the same team and were exposed to the same soil/muddy water, which may have harbored the spores. In addition, three cases were daily users of CLs and one case had history of ocular trauma that may have facilitated corneal infection. All our cases were men with a median age of 33 years, similar to previous case series [8, 17–19]. No case had any history of systemic diseases that could affect general health conditions. Also, all our cases had unilateral MKC as the main feature in otherwise healthy cases, contrasting with the bilateral infection seen in immunocompromised patients [17, 18]. In addition, all cases had diffuse conjunctivitis, sometimes associated with papillae as mentioned by other studies [5, 17, 18].

Due to the rarity of microsporidia as a cause of corneal infection and the lack of characteristic clinical examination findings, MKC appears likely to be frequently misdiagnosed. The multifocal epitheliopathy seen in MKC may be confused for Thygeson’s superficial punctate keratitis [20],*Acanthamoeba* keratitis [21], mycobacterial keratitis [16], or viral keratoconjunctivitis [17]. However, the multifocal, coarse, raised epithelial infiltrates seen in MKC are a key distinguishing feature from the above listed clinical entities.

AS-OCT and IVCM have been studied as alternative modes of diagnosis [5]. AS-OCT is a non-invasive, non-contact, high-resolution tool to obtain cross-sectional images of the cornea. The largest report included an observational study of 13 eyes, all of which were smear-positive for microsporidia spores [22]. The lesions are differentiated from adenoviral nummular scars as larger and sub-epithelial, with less intense hyperreflectivity and a smooth epithelial surface [23]. AS-OCT along with microbiological testing could facilitate diagnosis and help in monitoring treatment if validation studies could be undertaken [5]. Also, detection of hyper-reflective, pinpoint ovoid spores on IVCM offers a clue to MKC.

Microsporidia are not amenable to in vitro cultivation in routine culture media. As obligate intracellular organisms, microsporidia need a cell culture for growth, and this is not available in all laboratories. PCR is thus more suitable than tissue culture. PCR-based assays are useful for almost all types of microsporidial infections and can be applied to a wide range of clinical samples. Conners et al. described a PCR methodology that employs specific primers based on rRNA, followed by sequencing to identify microsporidia from corneal scrapings [24]. A multiplex PCR that simultaneously detects four species of microsporidia appears promising for use on ocular samples and can be adapted to diagnose MKC [25].

In the present study, PCR testing to diagnose human microsporidiosis identified *V. corneae* as the causative agent for all of the present cases. This is the first detection of *V. corneae* as a human infection in Japan. *V. corneae* (formerly known as *Nosema corneae*) has been detected in healthy individuals with keratoconjunctivitis (more than 300 cases associated with water or soil exposure) and human immunodeficiency virus-infected patients [26]. No natural host or reservoir in the environment has yet been identified for *V. corneae* and little evidence has been accumulated to explain why or how an environment becomes contaminated with *V. corneae.* The distribution of this pathogen in the environment thus needs to be defined.

No consensus has yet been reached regarding the optimal medical treatment for MKC and various therapeutic regimens have been attempted. Responses to medical therapies appear highly dependent upon the species involved and the immune status of the patient [20, 27]. Corneal debridement is not a new procedure; but is routinely performed for sample collection to allow microbiological diagnosis, and to reduce the microbial load. Large, dense white superficial punctate keratitis on the cornea could be entirely removed using a Bard-Parker blade #15, 26-gauge needle, or cotton swab, leaving behind a depression that heals without scarring [5, 28]. Conversely, a randomized clinical trial found no significant difference in final visual outcomes and resolution time between patients who did and did not undergo debridement; notably, none of the cases in that study received antimicrobial therapy [29].

Our cases recovered within few weeks after debridement and used SCL along with anti-microbial, anti-fungal, and anti-inflammatory eye drops. The ideal treatment regimen for microsporidial corneal infection remains unclear. This is particularly because MKC appears to represent a selflimiting epidemic keratoconjunctivitis in healthy individuals, invariably resolving without affecting vision [5, 12, 13, 19, 29, 30]. All cases in our case series recovered completely without any residual corneal opacifications and regained their normal BCVA.

Awareness of MKC has been growing in recent years, largely because of an increasing number of infections among healthy, immunocompetent individuals. Multifocal, coarse, punctate, unilateral keratitis associated with mild conjunctivitis appears to be the characteristic manifestation of this disease. Exposure to contaminated water or soil, in addition to inadequate sanitary facilities, represents a potential source of infection. Further investigations to clarify the characteristics of microsporidia seem warranted.

### Electronic supplementary material

Below is the link to the electronic supplementary material.

Supplementary Material 1

## Data Availability

The datasets used and/or analysed during the current study are available from the corresponding author on reasonable request. The data of DNA sequences was deposited in DDBJ with the number of (LC778281).

## List of abbreviations

AC: Anterior chamber
AIDS: Acquired immunodeficiency syndrome
AS-OCT: Anterior segment optical coherent tomography
BCVA: Best corrected visual acuity
CFW: Calcofluor White stain
DNA: Deoxyribonucleic Acid
FB: Foreign body
IRB: Institutional Review Board
IVCM: In vivo confocal biomicroscopy
Lt: Left
M: Male
MKC: Microsporoida keratoconjunctivitis
NA: Not applicable
PCR: Polymerase chain reaction
Rt: Right
SCL: Soft contact lens
