# Supplementary material for: Microsporidial keratoconjunctivitis – first outbreak in Japan
Source: BMC Infect Dis. 2023 Nov 1;23:752. doi: 10.1186/s12879-023-08767-y (PMC10621313; doi:10.1186/s12879-023-08767-y)
Supplement: Supplementary file 1 — Supplementary Material 1 [file 12879_2023_8767_MOESM1_ESM.pdf]

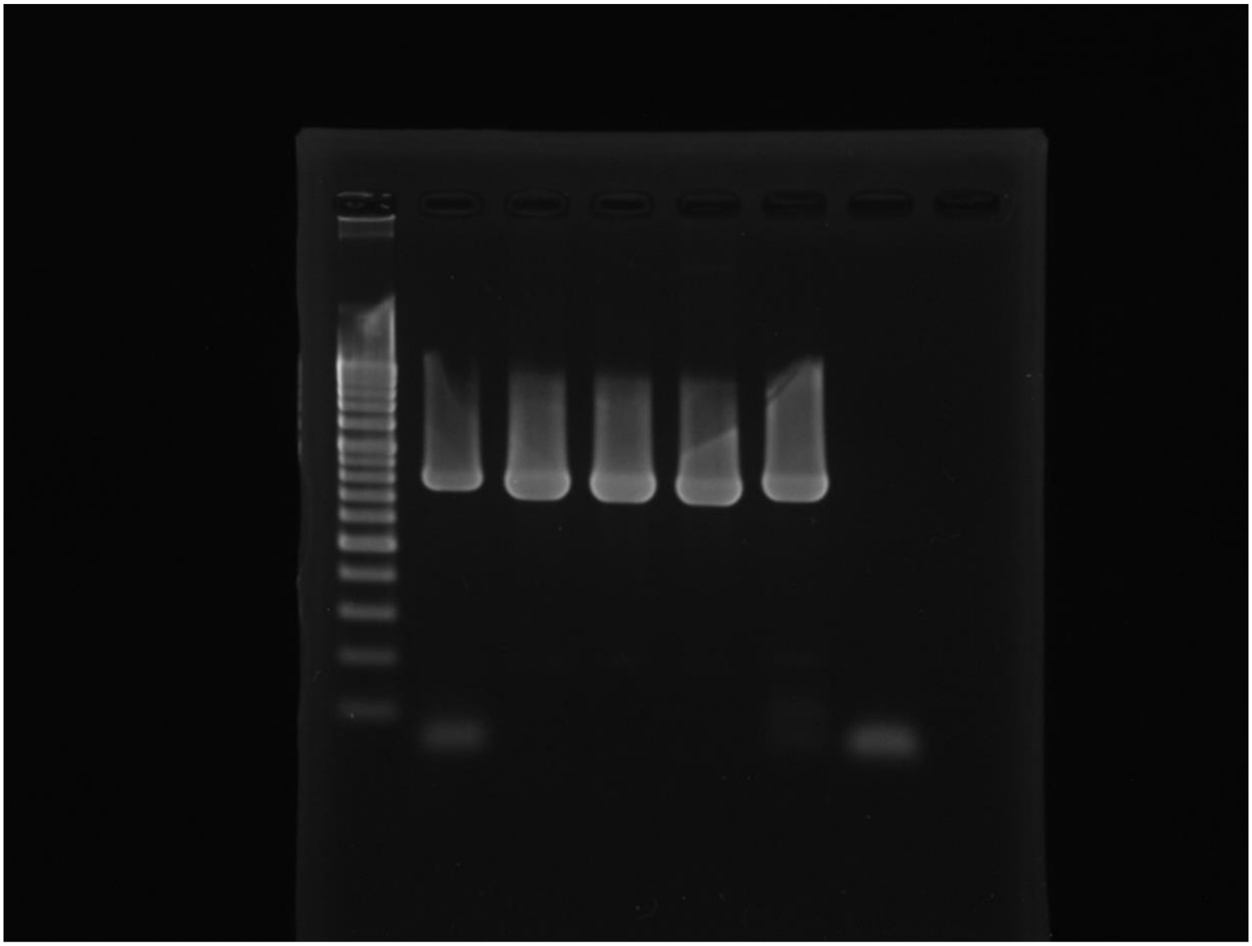

Figure Legend:

Supplementary figure of full-length gels and blots: Agarose gel electrophoresis of PCR products from DNA extracts of corneal scrapings. Lanes 1–4: case 2, case 3, case 4 and case 5, respectively; Lane 5: *Encephalitozoon cuniculi* DNA as a positive control; Lane 6: negative control. All four cases show PCR products of the same size, approximately 750 bp.
